# Lack of Bridge to Recovery in Pediatric Dilated Cardiomyopathy With Left Ventricular Noncompaction

**DOI:** 10.1016/j.atssr.2024.02.021

**Published:** 2024-08-22

**Authors:** Moyu Hasegawa, Masaki Taira, Yuji Tominaga, Takuji Watanabe, Yosuke Kugo, Toshiaki Nagashima, Akima Harada, Takayoshi Ueno, Shigeru Miyagawa

**Affiliations:** 1 Department of Cardiovascular Surgery, Osaka University Graduate School of Medicine, Yamadaoka, Osaka, Japan

## Abstract

**Background:**

This study assessed the possibility of a bridge to recovery using the Berlin Heart EXCOR and the histologic characteristics of pediatric patients with dilated cardiomyopathy accompanied by a left ventricular noncompaction phenotype.

**Methods:**

Of the 17 pediatric patients with dilated cardiomyopathy who underwent Berlin Heart EXCOR implantation between 2013 and 2020, 6 were diagnosed with left ventricular noncompaction association. The patients were classified into 2 groups: the dilated cardiomyopathy group and dilated cardiomyopathy with the left ventricular noncompaction phenotype group. The histologic characteristics of the left ventricular myocardium and left ventricular function after Berlin Heart EXCOR implantation were compared.

**Results:**

Cardiac recovery was not observed in the dilated cardiomyopathy with left ventricular noncompaction group. In contrast, 6 patients (55%) in the dilated cardiomyopathy group achieved cardiac recovery, and the Berlin Heart EXCOR was explanted. The degree of myocardial fibrosis was significantly higher in the dilated cardiomyopathy with the left ventricular noncompaction phenotype group than in the dilated cardiomyopathy group (*P* < .05). The final follow-up left ventricular ejection fraction and end-diastolic diameter during Berlin Heart EXCOR support improved significantly compared with the preimplantation variables in the dilated cardiomyopathy group (both *P* < .001); the dilated cardiomyopathy with left ventricular noncompaction phenotype group showed no improvements (*P* = .84 and *P* = .37, respectively).

**Conclusions:**

The left ventricular noncompaction phenotype associated with dilated cardiomyopathy may adversely affect the rate of cardiac recovery with Berlin Heart EXCOR.

**In Short**

▪ Association of the left ventricular noncompaction phenotype with dilated cardiomyopathy may adversely affect cardiac recovery with Berlin Heart EXCOR due to greater fibrosis and diastolic dysfunction.

We previously reported the bridge to recovery with Berlin Heart EXCOR (BHE) in pediatric dilated cardiomyopathy (DCM) and the associated histologic findings.^1^ Owing to the shortage of donors in Japan, we have routinely assessed the possibility of myocardial recovery 3 months after BHE implantation. However, there are still many unknowns in myocardial recovery cases, and the search for another clinical modality and bridge to recovery indicator is underway.

A recent study reported that some DCM cases are accompanied by left ventricular (LV) noncompaction (LVNC) and that children with DCM and LVNC had poor outcomes.^2^ LVNC is a rare cardiomyopathy characterized by prominent trabeculations in the LV and myocardium composed of 2 distinct layers of compacted and noncompacted tissue.^3^

In our pediatric patient group with DCM, echocardiography revealed that some patients had the LVNC phenotype. Therefore, this study aimed to review our experiences with pediatric patients with DCM accompanied by LVNC who underwent BHE implantation and compare the histologic characteristics with that of patients with DCM.

## Patients and Methods

### Ethics

The Osaka University Hospital Institutional Review Board reviewed and approved the study (approval number, 16105; approval date, Feb 11, 2016). Written informed consent was obtained from the legal guardian of each patient.

### Patients

**Figure 1:**
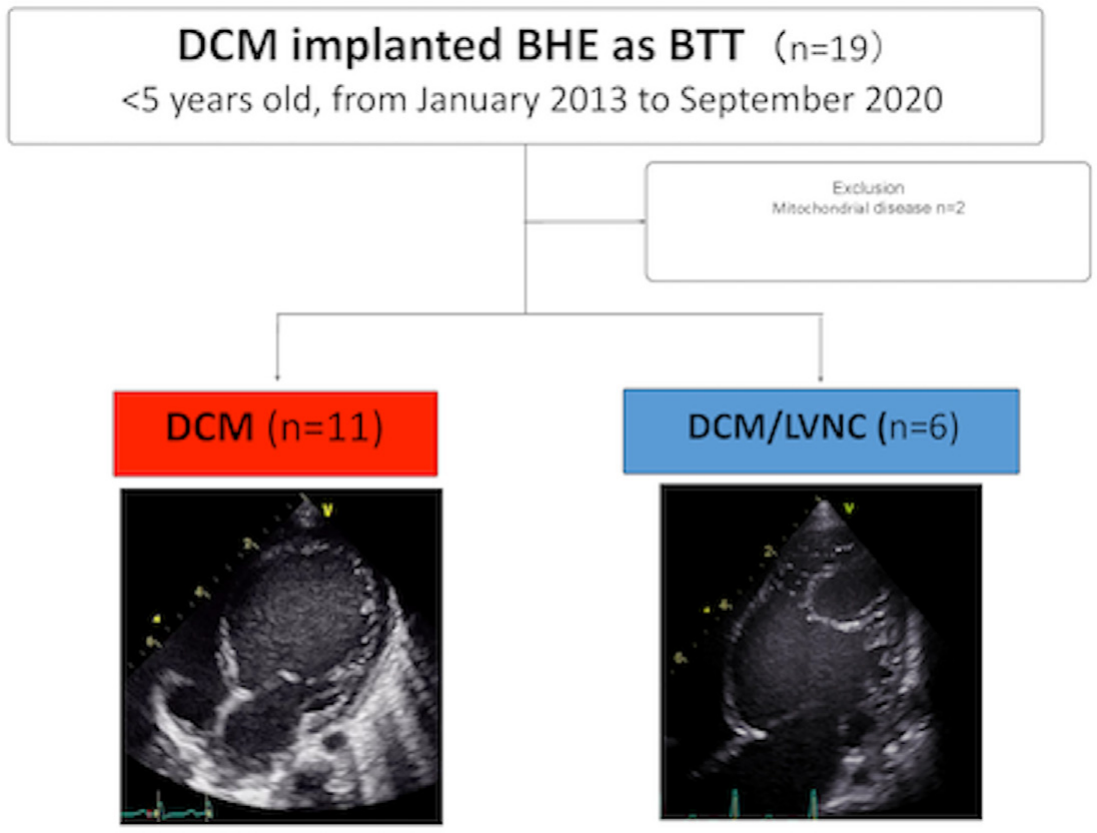
Overall, 11 patients were diagnosed with dilated cardiomyopathy (DCM) only (DCM group) and 6 were diagnosed with DCM accompanied by the left ventricular noncompaction (LVNC) phenotype (DCM/LVNC group) (BHE, Berlin Heart EXCOR; BTT, bridge to transplantation).

This retrospective study was conducted at the Osaka University Hospital. From 2013 to 2020, 19 patients aged <5 years who were diagnosed with DCM underwent BHE implantation in the LV as a bridge to transplantation. After 2 patients with mitochondrial diseases were excluded, 17 consecutive patients were included in this retrospective study. The indication for insertion of the BHE was severe heart failure unresponsive to intensive medical treatment with evidence of multiorgan failure. Diagnosis of LVNC was based on echocardiography. The diagnostic criteria were as follows: (1) prominent trabeculations, (2) 2 distinct layers composed of compacted and noncompacted myocardium, (3) noncompacted area in at least 1 segment, and (4) a noncompacted-to-compacted ratio >2 at end-systole. Overall, 11 patients were diagnosed with DCM only (DCM group), and 6 were diagnosed with DCM accompanied by the LVNC phenotype (DCM/LVNC group) (Figure 1).

### BHE Procedures and Clinical Protocol

The BHE implantation or explantation was performed under cardiopulmonary bypass on a beating heart. During implantation, LV myocardium was biopsied from the LV apex while the inflow cannula was inserted. The trabeculations of the LV were carefully resected before inserting the inflow cannula to prevent thromboembolism after implantation, particularly in the DCM/LVNC cases. After implantation, the pump flow was adjusted to 2.5 to 3.0 L/min/m^2^. The anticoagulation therapy protocol was acetylsalicylic acid combined with dipyridamole and warfarin. The treatment regimen for heart failure was carvedilol and angiotensin-converting enzyme inhibitors, which were gradually increased by 0.2 mg/kg/d, respectively.

### Assessment of Cardiac Function and Off-Test Protocol

Regular checkups, including echocardiography and cardiac catheterization, were performed at 1, 3, 6, and 12 months after BHE implantation. BHE explantation was considered after a minimum of 3 months of unloading using BHE and medical treatment in selected patients. During systemic heparinization (100 U/kg), the BHE pump rate was gradually decreased and then stopped. After 10 minutes of the heart beating without mechanical support, hemodynamic variables and LV function were assessed. Our explantation criteria were as follows: (1) LV ejection fraction (LVEF) >0.45, (2) LV end-diastolic diameter (LVDd) z score <+2, (3) pulmonary capillary wedge pressure (PCWP) <12 mm Hg, and (4) resting cardiac index >2.8 L/min/m^2^. If the patient cleared the off test, additional stress tests were conducted, such as water (10 mL/kg normal saline injection) and catecholamine (100 μg/kg/min noradrenalin administration)^2^ stress tests using similar off-test protocol.

### Histologic Studies

The LV myocardium specimens biopsied during the BHE implantation were fixed in paraffin and sectioned at a thickness of 5 μm. Subsequently, the section was stained with hematoxylin-eosin and Masson trichrome for light microscope examination.

The fibrosis area was assessed using MetaMorph 6.2 imaging software (Universal Imaging Corp, Downingtown, PA). The density of cluster of differentiation (CD) 31-positive vessels was assessed by counting the absolute number of microcapillaries (<20 μm) positive for CD31 antibody (ab28364; Abcam, Cambridge, UK) using the BZ-analysis software application (Keyence, Tokyo, Japan). The number of microcapillaries was divided by the number of cardiomyocytes in the same field, and the number of microcapillaries per cardiomyocyte was analyzed.

### Statistical Analysis

Statistical analyses were performed using JMP Pro 16 software (SAS Institute Inc, Cary, NC). Quantitative data are presented as mean (SD) or median (25th–75th percentile interquartile range [IQR]). Categorical variables were analyzed using the Pearson χ^2^ test. Continuous variables with normal distribution were analyzed using the Student *t* test after verifying the normal distribution using the Shapiro-Wilk test. The z-score of LVDd was calculated according to the recommendation by Lopez and colleagues.^4^ All statistical tests were 2-sided, and statistical significance was set at *P* ≤ .05.

## Results

### Patient Population

All patients were monitored for a median period of 4.6 years (IQR, 2.6-6.9 years) after BHE implantation. The Table summarizes the preimplantation patient characteristics.

**Table:** Preimplantation Patient Characteristics

Characteristic	DCM	DCM/LVNC	*P* Value
	(n = 11)	(n = 6)	
Male sex, n (%)	1 (9)	1 (17)	.33
Age at diagnosis, mo	5 (3–10)	3 (1–4)	.28
Left ventricular			
Ejection fraction	0.160 (0.063)	0.327 (0.115)	.002^a^
End-diastolic diameter, z-score	12.0 (3.9)	8.4 (3.9)	.11
PCWP, mm Hg	17.0 (6.9)	17.8 (3.8)	.82
Brain natriuretic peptide, pg/mL	2307 (1170–3234)	1763 (440–3499)	.51
Age at BHE implantation, mo	13.4 (5–13.5)	9.3 (4.5–11.5)	.52
Body weight at BHE implantation, kg	6.6 (2.9)	6.1 (0.9)	.66

Continuous data are presented as mean (SD) or median (interquartile range), and categorical data are shown as the n (%) of observations.

BHE, Berlin Heart EXCOR; DCM, dilated cardiomyopathy; LVNC, left ventricular noncompaction; PCWP, pulmonary capillary wedge pressure.

a Statistically significant (*P* ≤ .05).

**Supplementary Figure 1:**
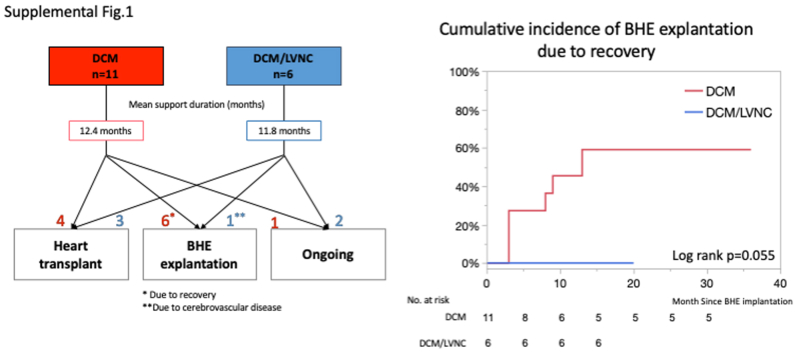


In the DCM group, 6 patients exhibited significant cardiac recovery and met the explantation criteria, undergoing successful BHE explantation after a median support period of 6.1 months (IQR, 3.7-10.6 months). All of these patients were discharged to home at a median of 5.9 months (interquartile range, 4.2-7.1 months) after explantation. To date, no heart failure recurrences or ventricular assist device reimplantations have been reported during an average follow-up of 62 months (IQR, 47.5-75.8 months) since discharge. Four patients underwent heart transplantation 10.7 to 37.2 months after BHE implantation. One patient remained on BHE support (Supplemental Figure 1).

In the DCM/LVNC group, 1 patient experienced a stroke 1 month after BHE implantation, and the BHE was explanted after 4.7 months of support. No patient in the DCM/LVNC group recovered cardiac function. Three patients underwent heart transplantation at 7.8 to 14.0 months after BHE implantation, and 2 patients remained on BHE support (Supplemental Figure 1).

### Cardiac Function After BHE Implantation

**Figure 2:**
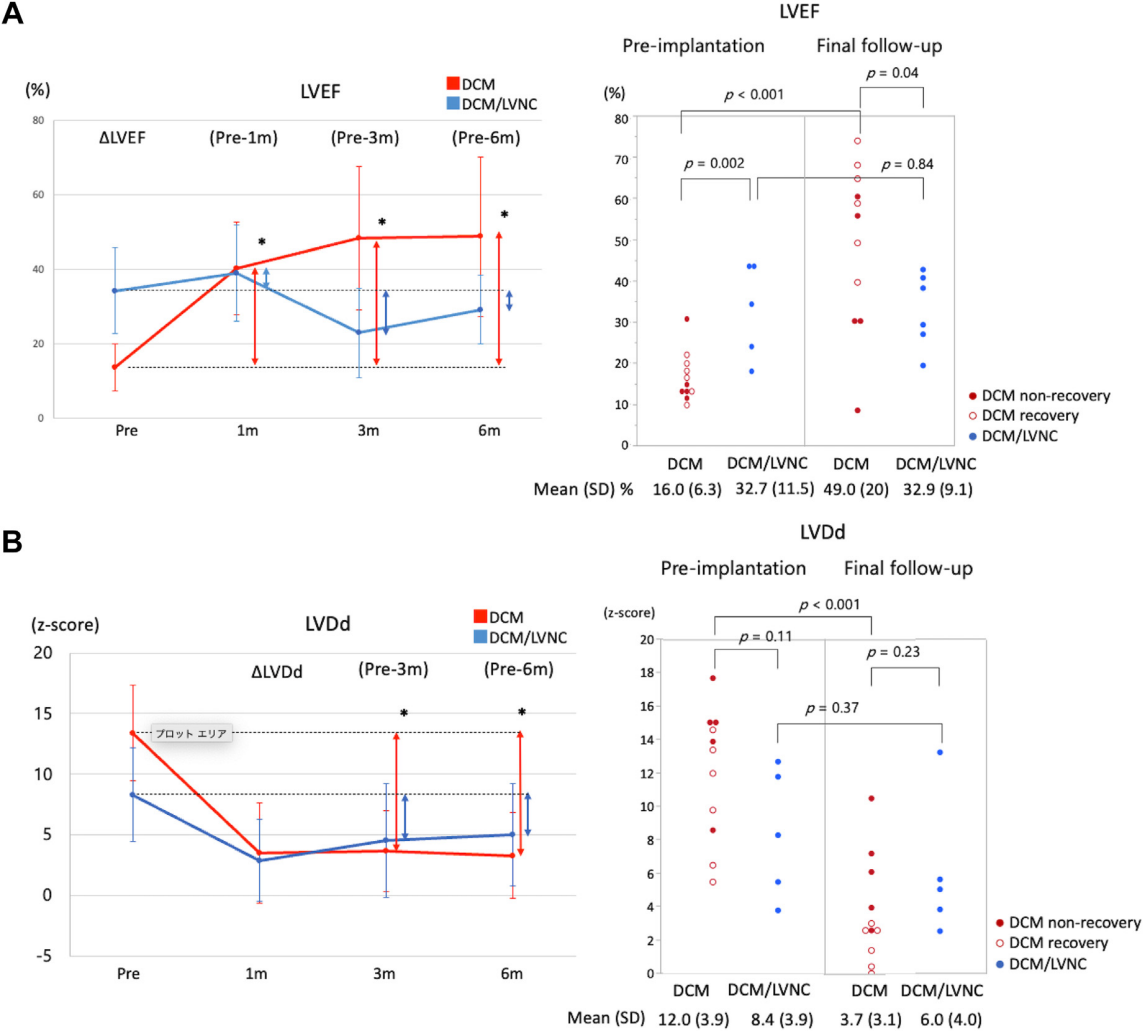
(A) The change in left ventricular ejection fraction (LVEF) over time after Berlin Heart EXCOR (BHE) implantation, and the LVEF at preimplantation and final follow-up during BHE support is shown. In the dilated cardiomyopathy/left ventricular noncompaction (DCM/LVNC) group, the LVNC did not improve during BHE support. (B) The change in the left ventricular end-diastolic diameter (LVDd) z-score over time after BHE, and the LVDd z-score of preimplantation and final follow-up during BHE support is shown. In the DCM/LVNC group, the LVDd did not improve during BHE support. ∗*P* < .05.

The time course and final follow-up for LVEF and LVDd are shown in Figure 2. Final follow-up examinations occurred before explantation or heart transplantation or were the most recent examination in ongoing cases. The final follow-up was performed at a median of 7 months (IQR, 3-10 months) after BHE implantation in the DCM group and 5 months (IQR, 5.3-6 months) in the DCM/LVNC group. The pre-LVEF in the DCM/LVNC group was significantly higher than that in the DCM group; however, the final follow-up LVEF was significantly lower (Figure 2). The LVEF in the DCM/LVNC group did not improve between preimplantation and the final follow-up.

The final follow-up LVDd z-score did not show a statistically significant difference between the DCM and DCM/LVNC groups. However, during BHE support, the LVDd z-score significantly decreased in the DCM group but not in the DCM/LVNC group.

**Supplementary Figure 2:**
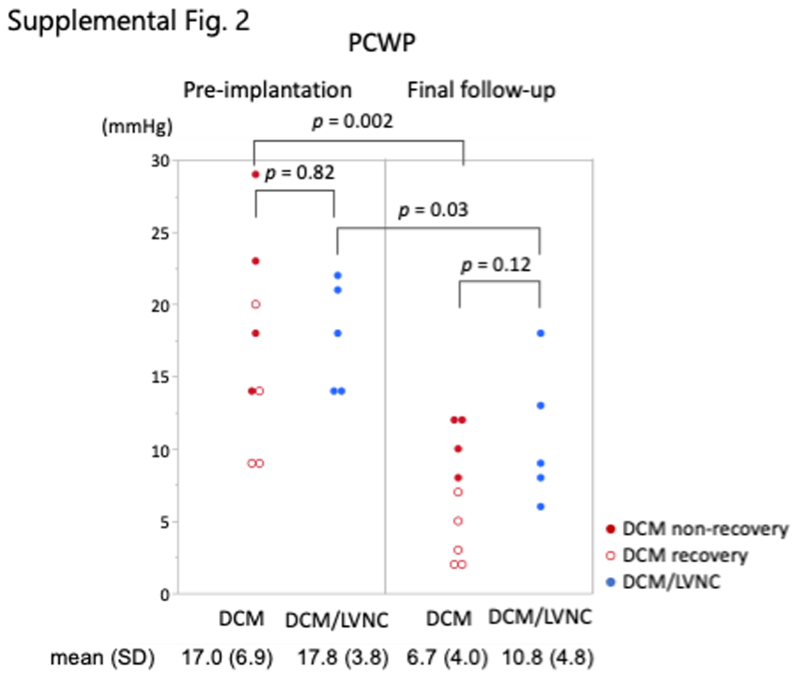


Regarding PCWP, which was measured during scheduled cardiac catheterization, the final follow-up PCWP tended to be higher in the DCM/LVNC group than in the DCM group (Supplemental Figure 2).

### Histologic Analysis

**Figure 3:**
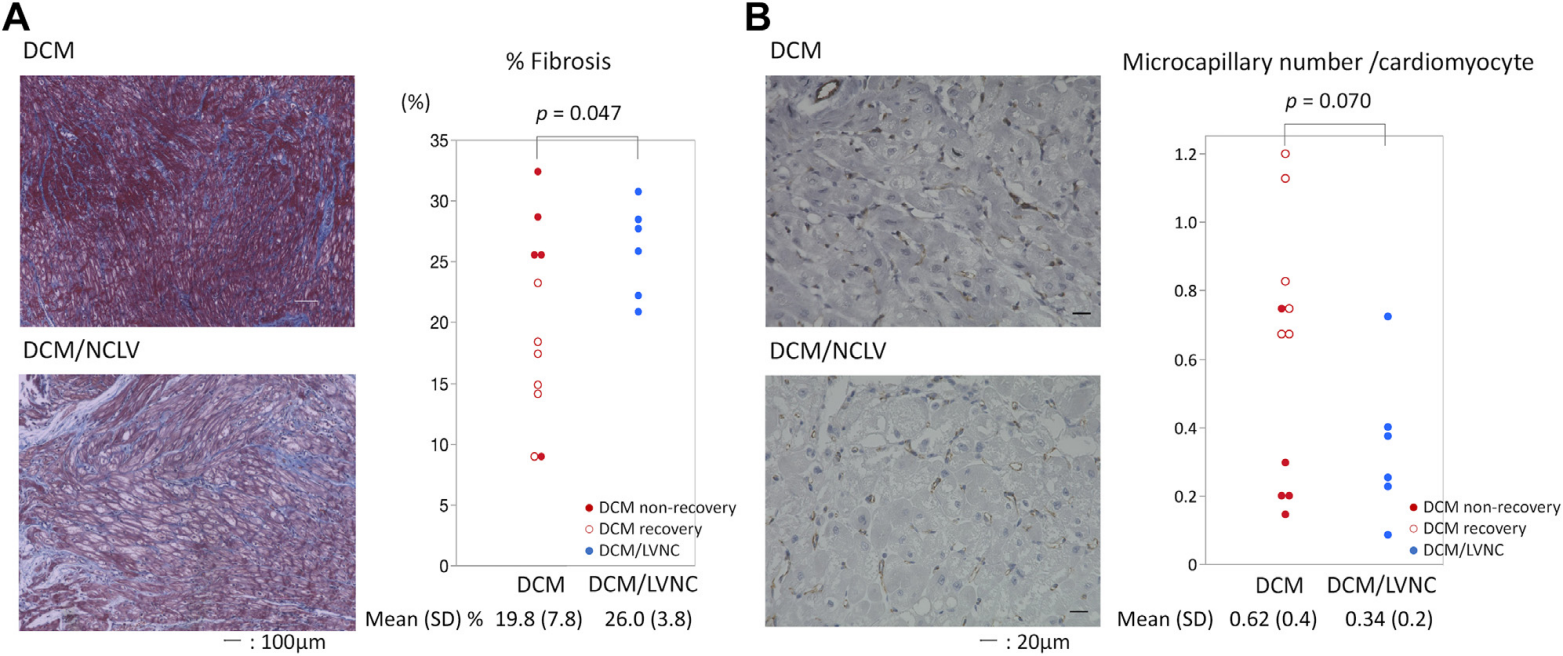
Histologic findings. (A) Representative photomicrographs (original magnification ×200, scale bar = 100 μm) of Masson trichrome staining at the midmural layer. The percentage of myocardial fibrosis was significantly higher in the dilated cardiomyopathy/left ventricular noncompaction (DCM/LVNC) group. (B) Representative photomicrographs (original magnification ×400, scale bar = 20 μm) of anti-cluster of differentiation 31 staining at the midmural layer.

The percentage of fibrosis during BHE implantation was 19.8% (SD, 7.8%) in the DCM group and 26.0% (SD, 3.8%) in the DCM/LVNC group. Fibrosis was significantly greater in the DCM/LVNC group than in the DCM group (*P* = .047) (Figure 3A). The number of microcapillaries per cardiomyocyte was 0.62 (SD, 0.4) in the DCM group and 0.34 (SD, 0.2) in the DCM/LVNC group (*P* = .070) (Figure 3B).

## Comment

LVNC is a rare cardiomyopathy; however, it is frequently associated with DCM.^2^ In our DCM group, 6 of 17 patients (35%) showed the LVNC phenotype, primacy. We report here the association between the LVNC phenotype and the cardiac recovery rate in pediatric patients with DCM and severe heart failure.

Remarkably higher LVEF, smaller LVDd, and higher PCWP were noted at preimplantation in the DCM/LVNC group than in the DCM group. This suggests that the cardiac dysfunction in the DCM/LVNC group was caused by a pathology of diastolic dysfunction rather than by contractile dysfunction, necessitating BHE implantation. Diastolic heart failure has been previously reported in a high-risk population for mechanical circulatory support,^5^ which may explain the low bridge to recovery rate.

Additionally, the high rate of explantation in the DCM group (55%) should be noted. This rate is higher in young patients with DCM associated with long-term BHE support than seen in a previous study.^6^ It is possible that in previous studies, the patients with associated LVNC phenotype were included among those diagnosed with DCM, as in our group. Our study suggests that a bridge to recovery may be far more possible with “pure” DCM.

Histologic characteristics of LVNC observed in an autopsied adult heart were previously reported as high fibrosis and subendocardial ischemia in the LV myocardium.^7^ Similar to the prior study, patients with DCM/LVNC showed greater fibrosis than those with DCM in our study. This LVNC’s potentially high degree of fibrosis could be associated with the decreased possibility of bridge to recovery with BHE in patients with DCM with the LVNC phenotype.

In conclusion, bridge to recovery with BHE implantation was not achieved in pediatric patients with DCM associated with the LVNC phenotype, who are at a higher risk of developing LV myocardial fibrosis and diastolic dysfunction. The LVNC phenotype associated with DCM may adversely affect the rate of cardiac recovery with BHE. However, this novel idea could still be used to investigate the bridge to recovery possibility based on the phenotype diagnosed before BHE implantation, and our study findings could be used to predict cardiac function recovery.

## References

[1] Tominaga Y., Ueno T., Kido T. (2020). Bridge to recovery with Berlin Heart EXCOR in children <10 kg with dilated cardiomyopathy: a histological analysis. Eur J Cardiothorac Surg.

[2] Brescia S.T., Rossano J.W., Pignatelli R. (2013). Mortality and sudden death in pediatric left ventricular non-compaction in a tertiary referral center. Circulation.

[3] Maron B.J., Towbin J.A., Thiene G. (2006). Contemporary definitions and classification of the cardiomyopathies: an American Heart Association Scientific Statement from the Council on Clinical Cardiology, Heart Failure and Transplantation Committee; Quality of Care and Outcomes Research and Functional Genomics and Translational Biology Interdisciplinary Working Groups; and Council on Epidemiology and Prevention. Circulation.

[4] Lopez L., Colan S., Stylianou M. (2017). Relationship of echocardiographic Z scores adjusted for body surface area to age, sex, race, and ethnicity: the pediatric heart network normal echocardiogram database. Circ Cardiovasc Imaging.

[5] Conway J., St. Louis J., Morales D.L.S., Law S., Tjossem C., Humpl T. (2015). Delineating survival outcomes in children <10 kg bridged to transplant or recovery with the Berlin Heart EXCOR Ventricular Assist Device. JACC Heart Fail.

[6] Hetzer R., Kaufmann F., Delmo Walter E.M. (2016). Paediatric mechanical circulatory support with Berlin Heart EXCOR: development and outcome of a 23-year experience. Eur J Cardiothorac Surg.

[7] Burke A., Mont E., Kutys R. (2005). Left ventricular noncompaction: a pathological study of 14 cases. Hum Pathol.

